# Fat and epidermal cell suspension grafting: a new advanced one-step skin regeneration surgical technique

**DOI:** 10.1186/1756-9966-33-23

**Published:** 2014-02-24

**Authors:** Emilia Migliano, Barbara Bellei, Flavio Andrea Govoni, Stefania Bucher, Mauro Picardo

**Affiliations:** 1 Department of Plastic and Reconstructive Surgery, San Gallicano Dermatologic Institute, IRCCS, Rome, Italy; 2 Laboratory of Cutaneous Physiopathology, San Gallicano Dermatologic Institute, IRCCS, Rome, Italy; 3 Department of Maxillofacial Surgery, San Filippo Neri Hospital, Rome, Italy

**Keywords:** Skin cancer sequelae, Lipofilling, Fat graft, Epidermal cell suspension, Skin graft, Dystrophic scar, Laser ablation, Cell therapy, Dermal revitalization

## Abstract

**Background:**

Dystrophic skin scarring commonly occurs following skin cancer resections. In particular, the cosmetic outcome of skin graft reconstructions, following epidermoidal carcinoma removal, is generally poor due to wide marginal tumour excision, loss of subcutaneous tissues, and subsequent pigmented atrophic scarring of the graft coverage. Skin grafting sequelae need a three dimensional correction to restore either the epidermal layer or the dermal/subdermal volume and vascularization.

**Methods:**

The surgeons combined CO_2_ laser ablation, subdermal lipofilling according to the Coleman’s technique and epidermal cell suspension autografting to correct wide depressed and dyschromic facial scar. The Authors applied this new technique on three nasal skin cancer resected patients: two of them actually need a longer follow-up, the third patient, a 48 yr old caucasian male, presented a skin grafting scar due to sclerodermiform basal cell carcinoma removal. This case is reported discussing pre-intra and post-operative records up to a complete twelve months follow-up.

**Results:**

Records at six and twelve months follow-up after surgery demonstrate a fully integrated skin graft and a good restoration of the treated area, presenting the same texture and pigmentation of the adjacent untreated skin. Optimal, stable three-dimensional skin cosmetic restoration was obtained in a single stage surgical procedure.

**Conclusion:**

Autologous non-cultured epidermal cell suspension transplantation on an epidermal laser ablated skin area, in combination with lipofilling subdermal reconstruction, appears to be an effective, simple and time-saving method to correct skin graft sequelae, in skin cancer patients. This new technique allows to restore a three-dimensional morphological structure of the treated area and to recover a natural appearance of the skin at the same time. The Authors believe that this technique can be safely used to treat any kind of dystrophic scarring.

## Introduction

Skin grafting reconstruction is widely used in patients who need surgical removal of cutaneous malignancies, but often leaves unpleasant, antiaesthetic and dystrophic scars. Skin grafting otherwise is mandatory either for oncological follow-up or for the presence of multiple precancerous lesions on the skin surrounding to the area that needs reconstruction. It is also used for wide defect coverage, especially in the facial region, where there are many areas of functional and cosmetic relevance that must be absolutely spared from flap surgery [1]. New techniques arising from regenerative medicine could be considered in reconstructive surgery, even in oncologic surgery, to improve treatment results and to obtain better aesthetic and cosmetic outcomes, using less invasive operations. Lipostructure (fat autografting performed via microcannulas) is a widely accepted surgical procedure for natural long-lasting tissutal volume restoration. This technique is frequently used to restore the morphological three-dimensional pattern of subdermal, hypodermal and muscular structures, where natural aging factors or pathological events have produced fat tissue loss or atrophy [2-4]. Skin tissue engineering using both cultured and non-cultured epidermal cells is currently applied for the treatment of chronic non-healing wounds [5,6] and stable vitiligo refractory to medical treatment [7-9]. Mechanical or physical dermabrasion (cryotherapic or laser epidermal ablation) are widely used to prepare the surgical field for the cellular suspension autografting. The combination of both surgical options, lipofilling and epidermal cellular grafting, has never been attempted before in the same procedure. The Authors have started a surgical trial of skin reconstructions combining these two techniques in order to evaluate if a multiplanar treatment can provide, in a single stage operation, better results if compared with the traditional treatments. This work is a preliminary report of a surgical trial actually in progress.

## Materials and methods

### Patient characteristics

**Figure 1:**
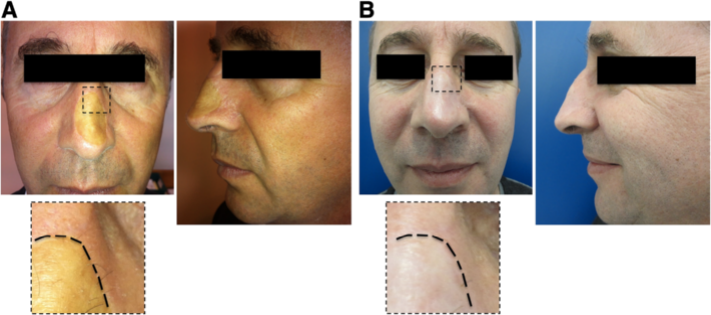
**First patient undergone one step surgical skin regeneration.** A 48 y.o. caucasian male presenting a wide (4×3 cm) depressed and dyschromic nasal skin-graft scar resulting from the resection of a sclerodermiform basal cell carcinoma. **A)** preoperative views, **B)** 12 months post-operative follow-up.

**Figure 2:**
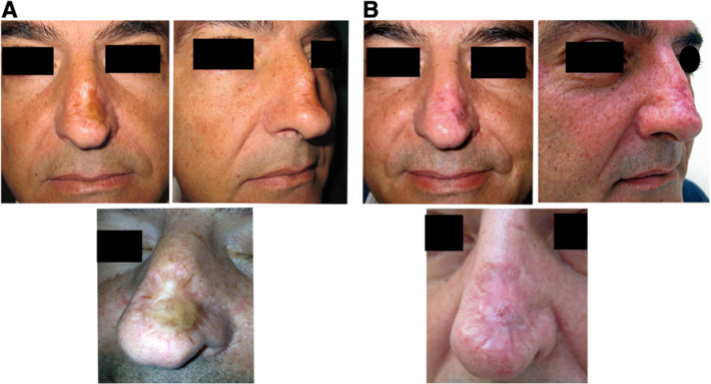
**Second patient undergone one-step surgical skin regeneration.** A 43 y.o. caucasian male, presenting a very similar skin graft scar sequela resulting from the resection of a sclerodermiform basal cell carcinoma. **A)** preoperative views, **B)** 1 month post-operative follow-up.

**Figure 3:**
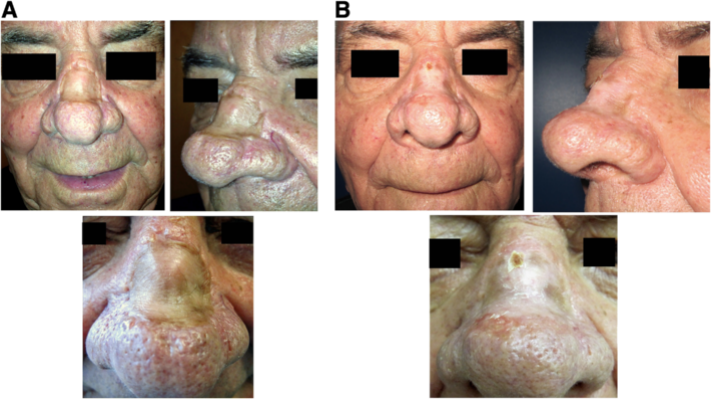
**Third patient undergone one-step surgical skin regeneration.** A 68 y.o. caucasian male, presenting a rhinophyma and very deep retracting skin graft scar of the nasal dorsum, resulting from the resection of a sclerodermiform basal cell carcinoma. **A)** preoperative views, **B)** 20 days post-operative follow-up.

Surgical trial selection criteria were: 1) nasal skin cancer resected patients (sclerodermiform basal cell carcinoma), 2) three years recurrence free follow-up, 3) wide nasal skin graft sequelae.At the time of publication three patients have been enrolled in this study (Figures 1,2,3). Two of them have a good but too short follow-up, in absence of immediate and short-term post-operative complications. The first patient enrolled in this study (Figure 1A), a 48 y.o. caucasian male, presented a wide (4×3 cm) depressed and dyschromic nasal skin-graft scar resulting from the resection of a sclerodermiform basal cell carcinoma. In the patient history, the wide resection and immediate skin graft reconstruction, occurred three years before, as an obliged treatment choice after two local recurrences of the skin cancer. All the patients enrolled in this study were extensively informed about technical details of the new procedure, they were informed also about risks and alternative surgical treatments. Written informed consent was obtained from all the patients for the publication of this report and any accompanying images. This new technique has been revised and approved as a reliable clinical research project by the I.F.O. Ethical Commitee, protocol n. 67/2012; the research is in compliance to the Helsinki declaration.

### Surgical technique

**Figure 4:**
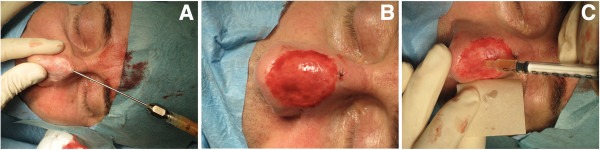
**Surgical technique: laser ablation, lipotransplantation and epidermal cell suspension graft.** Intraoperative views: **A)** the skin scarred area was prepared by a soft laser superficial ablation then fat injections have been performed using a spoon-tip blunt micro-cannula (1 mm). **B)** deeper Co_2_ laser ablation at the end of lipofilling prepared a bleeding dermal graft recipient site. **C)** epidermal non cultured cells were slowly dropped on the dermal bed (total volume of suspension dropped 1.3 ml).

1. A skin sample (0.5 cm × 0.5 cm) was taken from the post-auricular region under local anesthesia (2% lidocaine infiltration), resecting the skin in the superficial dermis. The donor skin was immersed in phosphate saline buffer and was transported to the cell biology laboratory to be processed as reported below.

2. Adipose tissue was harvested from the abdominal region using the Coleman’s technique (150 ml of Kleine’s solution infiltration). Ten minutes after the infiltration, a total of 40 ml of adipose tissue was syringe-suctioned with a 2-mm blunt cannula and collected in 10 ml syringes. The fat tissue was centrifuged for 3 minutes at 3000 rpm, then left in the aspiration syringes for at least 10 minutes to obtain a stable stratification in oil, fat tissue and blood/serum. The concentrated fat tissue (about 10 ml), purified from the oil and serum phase, was loaded in 1 ml syringes, using closed connection devices.

3. The skin scarred area was prepared to receive the cell suspension transplantation by an epidermal ablation, performed by a 2 W CO_2_ continuous laser beam (Smartoffice plus™ by DEKA-Italy) (Figure 4A), making attention to reduce vascular dermal damages. Dermal moderate bleeding is necessary to produce an adequate recipient bed for cellular implantation (Figure 4B). To obtain a better bed preparation the laser ablation has been fractioned in two phases: a) prelipofilling superficial ablation and b) deeper ablation after subdermal lipotrasplantation.4) Lipofilling has been performed, where it was possible, in a multiple layer stratification using a blunt micro-cannula (1 mm). The subdermal layer has been prepared, before fat filling, by a spoon tip 1 mm cannula over the deep perichondral nasal plane (Figure 4A). Total fat volume injected was approximatly of 10 ml. The treated area presented an average oval shape size of 4×3 cm.5. The epidermal non cultured cells were suspended in patient plasma in 1 ml syringes, then they have been slowly dropped on the dermal bed of the recipient site (total volume of suspension dropped 1.3 ml) (Figure 4C).

5. Wound nasal external dressing was applied using Veloderm™ (BTC S.r.l. Ancona-Italy) a special cellulose membrane, obtained through a biotechnologic process, patented as Cristalcell77™. The bio-membrane, that presents a good permeability, similar to normal human skin, was moistened with patient plasma.

### Laboratory phase

**Figure 5:**
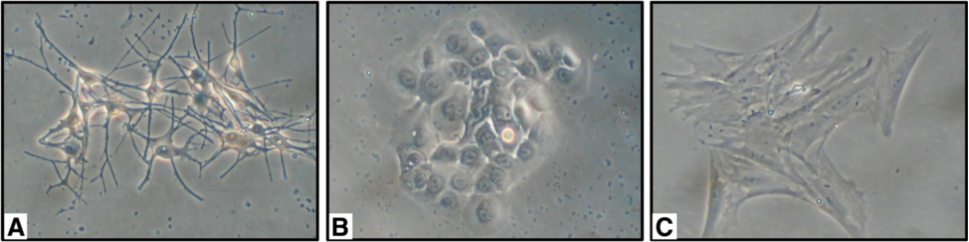
**Microscopic assay of epidermal cell suspension viability.** Microscopic observation of cell cultures. Melanocytes **(A)**, Keratinocytes **(B)** and Fibroblasts **(C)** were maintained in specific commercial culture medium and routinely observed under contrast microscope. Specific morphologic analysis confirmed the presence of epidermal cells and dermal fibroblast. The capacity to seed and to proliferate demonstrated that cell suspension contained mostly viable cells. Original magnification 20×.

1. Plasma preparation: patient plasma was obtained by collecting 7 ml of whole blood into heparin-treated tubes after centrifugation.

2. Preparation of single cell suspension: under sterile conditions, skin samples were broken into small pieces and incubated with 0.25% trypsin-0.05% ethylenediamine tetraacetic acid (EDTA) (Gibco BRL, Milan Italy) at 37°C for 30 min whilst the recipient site was prepared. In order to prevent digestion of separated cells, the reaction of trypsin-EDTA was stopped by adding one volume of patient plasma and cell suspension was then filtered through a 70-μm cell strainer (BD Bioscences, Milan Italy). Finally, the cell suspension was centrifuged for 5 min at 800 rpm to obtain a cell pellet, which was suspended in 0.4 ml of patient plasma. It was then transported to the operation theatre where the cell suspension was aspirated and drawn up into a clean syringe, ready for application. To monitor cell viability about 10% of cell suspension preparation was seeded into cell culture plates. Fibroblasts, keratinocytes and melanocytes were cultured separately for a week [8,9], morphological observations documented the presence of active replicating cells (Figure 5 A,B,C).

## Results

Five days after surgical treatment, all the medications were gently removed by 0.9% NaCl solution moistening. At the time of the first medication the cell graft demonstrated to be well integrated, in all patients. Veloderm™ membranes have been applied once more at medication time, on all the grafts for seven days more. At the second medication, twelve days after the surgical treatment, the grafts were fully integrated and the treated areas were unnoticeable if compared to the surrounding untreated skin. Only in one patient a small area (about 2 mm) in the peripheral region lightly bleeding, was successfully treated with a zinc oxide moisturizer. All the patients were encouraged to use maximum sunscreen protection for at least six months. The patient actually in full follow-up was examined and photo-recorded six and twelve months after surgery. The treated area appeared normally reepithelizated showing the same texture and pigmentation as the adjacent untreated skin (Figure 1B). Photographic and clinical measurements demonstrated that the injected subdermal fat resorption rate was minimal as expected. Photo shots of pre and postopearative short-term follow-up records of the other two cases enrolled in this preliminary study are reported in Figures 2 and 3.

## Discussion

Forehead frontal flap should be a good surgical alternative technique for the removal of large nasal dorsum scars. However it produces new wide frontal scars, and requires more surgical times to obtain optimum results [10,11]. The upcoming techniques used in cosmetic surgery seem to be really promising for correcting scars in a better way than traditional flap surgery.

Considering that our Institute has a growing experience in tissue regeneration techniques [8,9], we have planned to combine lipoaspirate transplantation with non-cultured cell-based therapy. The technique that we have described associates, for the first time in a single surgical stage, the lipofilling for the volumetric correction of scar atrophy to the transplantation of keratinocytes and melanocytes for the revitalization and repigmentation of the epidermal layers. The combination of the two techniques could lead to a synergistic effect in the enhancement of cell grafts results, in a time and costs saving procedure.

The use of adipose tissue for transplantation in plastic surgery dates back to 19^th^ century [12]. Illouz described cases of fat grafting using cannulas for aspiration and injection [13], Guerrerosantos implanted mini-fat grafts to correct patients affected by Parry-Romberg syndrome, and to improve facelift results [14]. Similar successful results were reported in facial aesthetic surgery, by may Authors, in terms of improvement of the three dimensional facial outlook, as well as decreasing both recovery time and post-operative complications. One of the critical points outlined by all Authors is the fragility of human adipose tissue. All Authors have reported in fact an high rate of postoperative fat resorption. In 1995 Coleman [15] introduced new advanced lipotransplantation techniques reducing the manipulation of fat tissue at a bare minimum. Coleman’s method [2,3] consists in the use of small blunt cannulae to reduce the damage of adipocytes during the aspiration phase, in combination with the use of a closed centrifugation system to concentrate fat pads, removing free oils, infiltrate solution, and blood at the same time. In the injection phase of fat transplantation Coleman suggested to use small cannulas, to create subdermal and hypodermal multiple tunnels, releasing only small amounts of fatty tissue in the recipient area, using a multilayer technique of implantation. This method has been proven to reduce fat damage and to improve three-dimensional fat distribution. The consequent reduction of adipocyte necrosis and the improvement of graft vascularity is probably the key-point that explains the long lasting results obtained.

Refined fat injection-manipulation procedures strongly benefit also to adult adipose tissue stem cells, stromal stem cells, contained in the transplanted tissues, that can stimulate growth and angiogenetic factors release [4,16]. All these components could also play a relevant role during the epidermal cell suspension graft. In this regard, the autologous transplanted fat tissue, not only corrects appropriately facial depressions, but also offers a natural source of nutrients and vascular growth factors to the overlaying dermal tissues [15].

The grafts of epithelial cell suspensions (cultured or non-cultured) have generated interest due to the broad-spectrum of applications such as severe burns, chronic non-healing wounds, vitiligo, and reconstruction after excision of giant congenital nevi [5-7,17,18]. These transplantation techniques make easier the choice of an adjacent skin donor site and greatly reduce the amount of skin to be resected for cell preparation, if compared to other procedures. Moreover, skin substitutes, including autologous cultured cells, are markedly expensive [18], whereas non-cultured autologous epidermal cell suspensions can be low cost prepared in a relatively short time, during the same surgical operation. Nevertheless, this therapeutic approach is still rarely applied in modern clinical practice. In this experimentation, we modified the standard protocol by adding autologous plasma as a carrier for keratinocyte-melanocyte cell suspension instead of the defined chemical cell medium. Plasma components, especially dissolved proteins and hormones, act as a natural source of growth factors and essential nutrients for grafted cells.

The preparation of the receiving site by a CO_2_ laser resurfacing if compared to mechanical dermabrasion is more accurate in sampling the depth with an easily affordable post-operative course. This method seems also to improve cellular adhesion and survival. The dressing with an interactive cellulose bio-membrane as a provisional epidermal substitute (Veloderm™), frequently used for the treatment of difficult wounds and burns, offers the advantage to create the ideal microenvironment for optimal re-epithelization and wound infection prevention.

Cancer surveillance can be better guaranted using cell transplantation combined to the lipofilling technique where improvement in volume, mini-invasive skin scar debridement, and better vascularization can be obtained without moving the surrounding skin flaps.

The risk of skin graft and cartilage necrosis was prevented by a percutaneous multilayer gentle debridment of the recipient site obtained by 1 mm spoon-tip microcannula before fat injection. This subdermal and extraperichondral pre-grafting soft tunnelization reduces the postoperative skin graft ischemia, lowering at the same time fat graft resorption rate. A recent article by Nguyen and Magalon demonstrated that microfat injections, performed by 0.8 mm microcannula in a mouse model of dermal fibrosis, allow better skin graft revascularization [19]. This hypothesis may possibly explain the improvement of the results observed in our cases of epidermal cell suspension combined to lipofilling, if compared to vitiligo patients treated in our Institute, without concurrent subdermal grafting. Our preliminary observation is confirmed also from Daumas and Magalon who reported encouraging results in Leukoderma obtained through subdermal fat grafts [20].

The results obtained in our first patient were stable at 12 months and did not require any further fat volume filling, demonstrating also good trophic effects on the dermis of the skin grafted area.

In 1992 Humbley and Carruthers described four clinical cases of nasal depressed scars treated by fat lipofilling, reporting persistent excellent results. They recommended to use minimally invasive subdermal dissection technique and where possible to correct large depressions repeating two or three times the grafting procedures, to prevent fat resorption and skin necrosis [21].

In our opinion the combination of lipofilling with epidermal cell suspensions, transferred in autologous plasma, showed very good results if compared to those expected from separate procedures. Anyway we can’t demonstrate, with this preliminary report, if the results we have obtained, could be really superior to traditional procedures. We are convinced empirically that lipoinjections can produce a revitalization and revascularization of the atrophic scarred dermis, enhancing the engraftment of the epidermal cells [22-24]. These clinical observations naturally have to be statistically demonstrated on a larger sample of patients. Finally we have to mention that cost expenses of the procedures used in this trial are low and affordable, in particular they don’t require special commercial devices or prefabricated cellular preparation kits.

## Conclusions

The Authors report three successful cases of simultaneous lipofilling and epidermal cell suspension grafting for the treatment of skin graft sequelae, in nasal wide cutaneous cancer resected patients. The combination of this two techniques, despite of the lack of scientific evidence in the literature, allowed the simultaneous correction of nasal depression and the restoration of a dyschromic/dystrophic skin coverage. The results obtained demonstrated to be stable at the 12 months follow-up with an evident good unexpected trophic effect on the dermis of the skin grafted area. The cell therapy used is cost effective as well as the lipotransplantation procedures. It provided faster skin reconstruction, in a one-stage minimally invasive surgical operation, sparing healthy adjacent skin, obtaining good and stable cosmetic results with faster clinical recovery, if compared to traditional flap surgery.

## Competing interests

The authors declared that they have no competing interests.

## Authors’ contributions

EM was the research leader, conceived the study, performed surgical operations, drafted and revised the manuscript. BB and MP partecipated in conceiving the study and performed all the laboratory phases. FAG performed a critical revision of the research and partecipated to the final manuscript revision. SB contributed to the financial support of the research and were involved in the final approval of the manuscript. All the authors read and approved the final manuscript.

## References

[B1] Cocke WM The free graft: its value in reconstruction after operation for head and neck cancer Am Surg 1976 42 3 223 226 769621

[B2] Coleman SR Facial recontouring with lipostructure Clin Plast Surg 1997 24 347 367 9142473

[B3] Coleman SR Structural fat grafting: more than a permanent filler Plast Reconstr Surg 2006 118 108S 120S 10.1097/01.prs.0000234610.81672.e7 16936550

[B4] Folgiero V Migliano E Tedesco M Iacovelli S Bon G Torre ML Sacchi A Marazzi M Bucher S Falcioni R Purification and characterization of adipose-derived stem cells from patients with lipoaspirate transplant Cell Transplant 2010 19 1225 1235 10.3727/09638910X519265 21208530

[B5] Shukla VK Tiwary SK Barnwal S Gulati AK Pandey SS Effect of autologous epidermal cell suspension transplantation in chronic non-healing wounds: a pilot study Can J Surg 2010 53 6 10 20100406 PMC2810008

[B6] Zweifel CJ Contaldo C Köhler C Jandali A Künzi W Giovanoli P Initial experiences using non-cultured autologous keratinocyte suspension for burn wound closure J Plast Reconstr Aesthet Surg 2008 61 e1 e4 17869200 10.1016/j.bjps.2007.07.015

[B7] El-Zawahry BM Zaki NS Bassiouny DA Sobhi RM Zaghloul A Khorshied MM Gouda HM Autologous melanocyte-keratinocyte suspension in the treatment of vitiligo J Eur Acad Dermatol Venereol 2011 25 215 220 10.1111/j.1468-3083.2010.03759.x 20569286

[B8] Bellei B Mastrofrancesco A Briganti S Aspite N Ale-Agha N Sies H Picardo M Ultraviolet A induced modulation of gap junctional intercellular communication by p38 MAPK activation in human Keratinocytes Exp Dermatol 2008 17 115 124 10.1111/j.1600-0625.2007.00662.x 18047584

[B9] Bellei B Pitisci A Ottaviani M Ludovici M Cota C Luzi F Dell’Anna ML Picardo M Vitiligo: a possible model of degenerative diseases PLoS One 2013 8 e59782 10.1371/journal.pone.0059782 23555779 PMC3608562

[B10] Menick FJ Nasal reconstruction with a forehead flap Clin Plast Surg 2009 36 3 443 459 10.1016/j.cps.2009.02.015 19505613

[B11] Menick FJ Aesthetic and reconstructive rhinoplasty: a continuum J Plast Reconstr Aeshet Surg 2012 65 9 1169 1174 10.1016/j.bjps.2012.04.017 22554677

[B12] Neuber F Fettransplantation. Bericht über die Verhandlungen der Dt Ges Chir Zentralbl Chir 1893 22 66 66

[B13] Illouz YG Present results of fat injection Aesthetic Plast Surg 1988 12 175 181 10.1007/BF01570929 3189036

[B14] Guerrerosantos J Simultaneous rhytidoplasty and lipoinjection: a comprehensive aesthetic surgical strategy Plast Reconstr Surg 1998 102 191 199 10.1097/00006534-199807000-00032 9655427

[B15] Coleman SR Long-term survival of fat transplants: controlled demonstrations Aesthetic Plast Surg 1995 19 5 4a 21–5 10.1007/BF00453875 8526158

[B16] Zuk PA Zhu M Ashjian P De Ugarte DA Huang J Mizuno H Human adipose tissue is a source of multipotent stem cells Mol Biol Cell 2002 13 4279 4295 10.1091/mbc.E02-02-0105 12475952 PMC138633

[B17] Mysore V Salim T Cellular grafts in management of leucoderma Indian J Dermatol 2009 54 142 144 10.4103/0019-5154.53194 20101310 PMC2807154

[B18] O’Neill TB Rawlins S Wood F Treatment of a large congenital melanocytic nevus with dermabrasion and autologous cell suspension (ReCELL®): a case report J Plast Reconstr Aest Surg 2011 64 1672 1676 10.1016/j.bjps.2011.05.016 21664206

[B19] Nguyen PS Desouches C Gay AM Hautier A Magalon G Development of microinjection as an innovative autologous fat graft technique: the use of adipose tissue as dermal filler J Plast Reconstr Aesthet Surg 2012 65 1692 1699 10.1016/j.bjps.2012.06.014 22749704

[B20] Daumas A Eraud J Hutier A Sabatier F Magalon G Granel B Potentialités and potentials of adipose tissue in scleroderma Rev Med Interne 2013 S0248–8663 13 630 639 10.1016/j.revmed.2013.08.007 24050783

[B21] Hambley RM Carruthers JA Microlipoinjection for the elevation of depressed full-thickness skin grafts on the nose J Dermatol Surg Oncol 1992 18 11 963 968 10.1111/j.1524-4725.1992.tb02768.x 1430553

[B22] Kouri RK Smit JM Cardoso E Pallua N Lantieri L Mathijssen IM Kouri RK jr Rigotti G Percutaneous Aponeurotomy and Lipo-Filling (PALF)- a regenerative alternative to Flap Reconstruction? Plast Reconstr Surg 2013 132 5 1280 1290 10.1097/PRS.0b013e3182a4c3a9 23924652

[B23] Coleman SR Mazzola RF Fat injection From filling to regeneration, Volume Chapter 11, 16 2009 II QMP St. Louis, Missouri: Quality Medical Publishing INC

[B24] Larocca RA Moraes-Vieira PM Bassi EJ Semedo P de Almeida DC Burgos da Silva MT Thornley T Pacheco-Silva A Saraiva Camara NO Adipose tissue derived mesenchymal stem cells increase skin allograft survival and inhibit Th-17 immune response Plos One 2013 8 10 e76396 doi:10.1371/journal.pone.0076396. eCollection 2013 10.1371/journal.pone.0076396 24124557 PMC3790669

